# Spontaneous Tumor Lysis Syndrome in the Setting of Pancreatic Adenocarcinoma

**DOI:** 10.7759/cureus.72735

**Published:** 2024-10-30

**Authors:** Brendan M Coyne, Syed M Ishaq, Cameron J Coyne, Brandon S Shiflett, Joshua T Birnbaum

**Affiliations:** 1 Internal Medicine, George Washington University School of Medicine and Health Sciences, Washington, USA; 2 Medicine, Sinai Hospital of Baltimore, Baltimore, USA; 3 Biology, University of California Riverside, Riverside, USA; 4 Internal Medicine, Sinai Hospital of Baltimore, Baltimore, USA

**Keywords:** acute spontaneous tumor lysis syndrome, critical care and internal medicine, ductal pancreatic adenocarcinoma, electrolyte imbalance, gastro-hepatic-pancreato-biliary disorders, metabolic abnormalities, oncologic emergencies, oncology and critical care, pulmonary metastasis of pancreatic cancer, tumor lysis syndrome

## Abstract

Tumor lysis syndrome (TLS) is the destruction of tumor cells, causing the release of intracellular contents, including but not limited to uric acid, phosphate, and potassium. This can manifest with significant physiologic abnormalities, such as life-threatening acute kidney injury. Although TLS is usually caused by the initiation of cytotoxic chemotherapy, it may also occur spontaneously, particularly in high-grade malignancies with high tumor burden and/or rapid cell turnover. Here, we present a case of spontaneous TLS in a patient with early-phase pancreatic cancer diagnosis and possible signs of metastasis. Additionally, we provide a comprehensive review of the current literature regarding this phenomenon. In the present case, our patient was stabilized using aggressive intravenous hydration and pharmacotherapy. This case report adds to the slowly growing literature surrounding the diagnosis and management of spontaneous TLS in pancreatic adenocarcinomas.

## Introduction

Tumor lysis syndrome (TLS), defined as the release of intracellular contents upon tumor cell destruction, is the most common oncologic emergency [1]. Cancers that are at the highest risk for TLS include high-grade hematological malignancies with rapid cellular turnover, such as Burkitt lymphoma and acute lymphoblastic leukemia [2]. TLS is usually caused by the initiation of cytotoxic chemotherapy, although it may occur spontaneously as well, particularly in tumors with a high proliferative rate or large tumor burden [1]. Spontaneous TLS is an uncommon complication of solid tumors; however, it has occurred in certain cases such as metastatic hepatic carcinoma, cholangiocarcinoma, and gastric adenocarcinoma [3-5]. Spontaneous TLS has been observed in pancreatic adenocarcinoma as well, and, as with other cancers, TLS appears to occur more often in advanced and/or metastatic disease [6-10]. When diagnosed early, the prognosis of TLS overall is favorable; however, the prognosis of spontaneous TLS is unfortunately usually poor, with especially high mortality in solid tumors [11]. Because of its severity, it is crucial to identify and initiate treatment early for spontaneous TLS. This report details a rare case of spontaneous TLS in pancreatic adenocarcinoma, with early identification and improvement of the metabolic abnormalities.

## Case presentation

A 75-year-old male with a past medical history of hypothyroidism, left tonsillar cancer in 2019 status post-chemoradiation, right carotid artery occlusion status post-stent, and recently diagnosed pancreatic adenocarcinoma initially presented to our hospital for worsening dehydration and inability to eat. He was found to have labs suspicious for TLS with acute kidney injury (AKI), including creatinine (Cr) of 3.95 mg/dL (baseline: 0.5 mg/dL), potassium of 5.5 mmol/L, uric acid of 10.8 mg/dL, phosphate of 12.3 mg/dL, and calcium of 5.9 mg/dL (Table 1), prompting admission to the intensive care unit before step-down to the intermediate care unit.

**Table 1:** Laboratory results. *Only one uric acid level was measured on admission. LDH measurements were not repeated by discharge. LDH: lactate dehydrogenase; BUN: blood urea nitrogen

Laboratory result (reference range)	Units	Admission	Discharge
Sodium (135–145)	mmol/L	126–128	130–133
Potassium (3.5–5.0)	mmol/L	5.5–5.8	3.4–3.8
Chloride (95–105)	mmol/L	84–91	84–86
Bicarbonate (23–30)	mmol/L	12.2–16.7	30.4–30.7
Anion gap (4–12)	mmol/L	11.9–12.3	17–18
Uric Acid (3.5–7.2)	mg/dL	10.8*	4.7–5.1
Calcium (8.5–10.5)	mg/dL	5.0–6.0	6.5–6.6
Phosphate (2.5–4.5)	mg/dL	11.9–12.3	6.9–7.3
Bilirubin (0.1–1.2)	µmol/L	19.9	13.8
LDH (125–220)	µ/L	259	-
BUN (10–20)	mg/dL	93–148	62–151
Creatinine (0.7–1.3)	mg/dL	3.3–3.95	4.56–4.61

**Figure 1:**
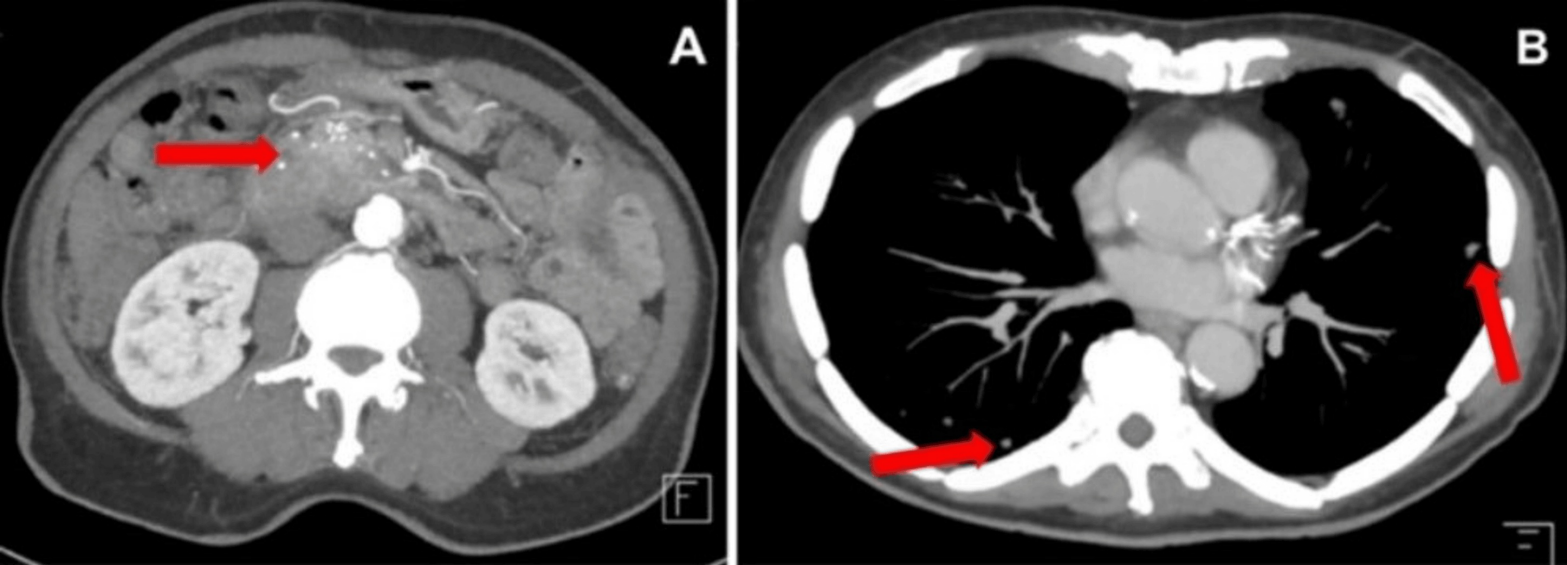
Patient’s initial CT imaging with contrast. (A) CT scan of the abdomen demonstrating marked biliary dilatation, an overdistended gallbladder, and a 3.8 × 2.1 cm mass in the head of the pancreas (arrow). (B) CT scan of the thorax demonstrating multiple pulmonary nodules concerning for possible metastasis (arrows).

**Figure 2:**
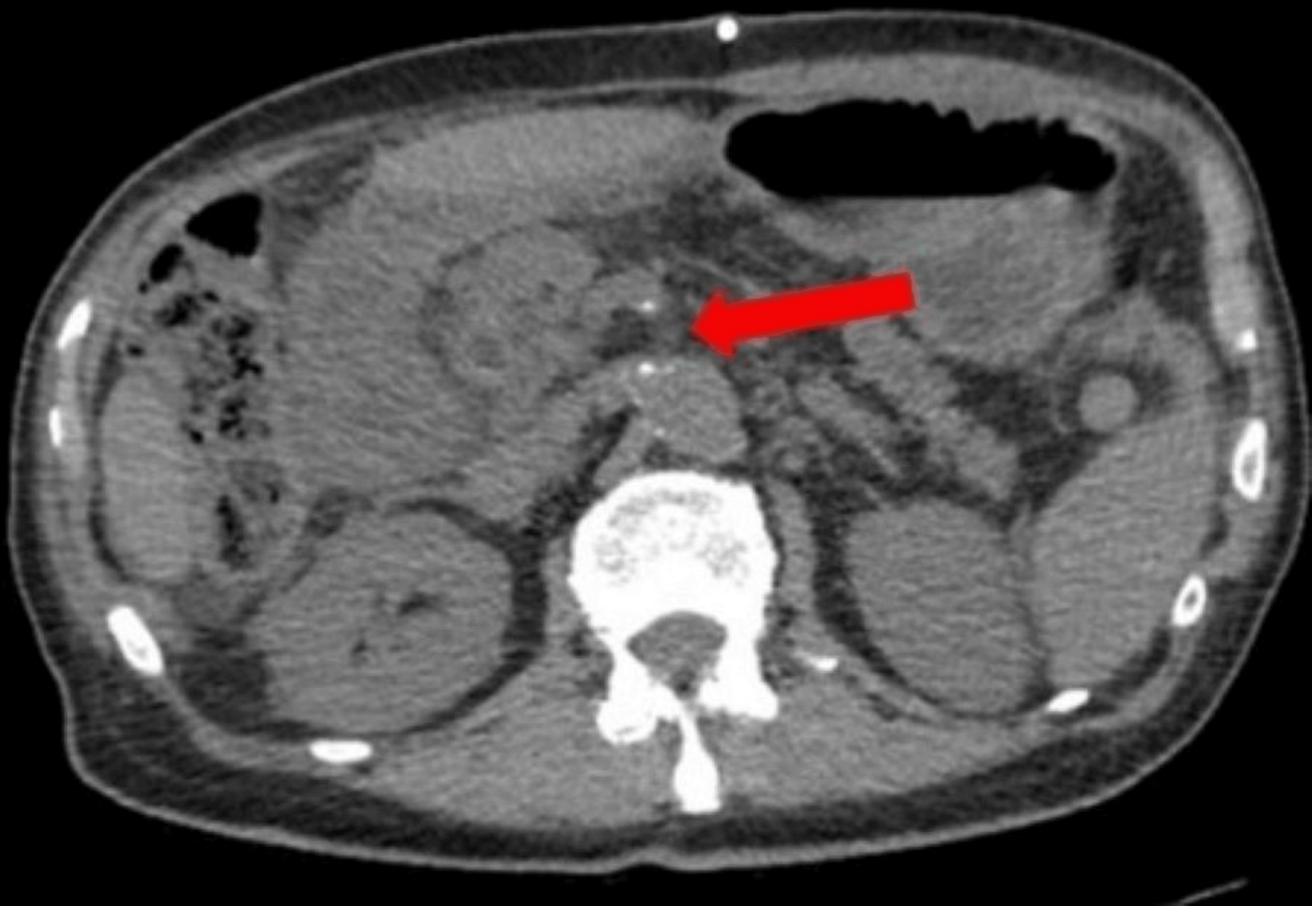
Repeat CT of the abdomen without contrast. Image showing a grossly similar appearance of the pancreatic tumor (arrow). Mildly prominent pancreatic lymph nodes and dilatation of the extrahepatic biliary, intrahepatic biliary, and pancreatic ducts can be visualized.

The patient underwent computed tomography (CT) imaging without contrast of his abdomen, pelvis, and neck. He had recently received CT imaging with contrast two weeks before presentation, which showed a 3.8 × 2.1 cm mass in the pancreatic head (Figure 1A). Numerous irregular lung nodules were also visualized in the bilateral lung bases, which were concerning for possible pulmonary metastasis, although they were not confirmed by biopsy (Figure 1B). On the patient’s repeat CT from the current admission, these pulmonary nodules slightly increased in size. Due to the non-contrast nature of the current CT, the pancreatic tumor was poorly visualized; however, it appeared grossly similar to his previous scan (Figure 2).

For the patient’s TLS and AKI, he received 1000 mL of intravenous (IV) normal saline (0.9% NaCl) before starting continuous IV dextrose 5% in water (D5W) with 150 mEq of NaHCO_3_. He was also given one 3 mg dose of IV rasburicase for his hyperuricemia and was started on 800 mg of PO sevelamer for his hyperphosphatemia. The patient exhibited marked improvement in his TLS labs on his treatment regimen (Table 1). His hyperuricemia, hyperkalemia, hyperphosphatemia, and acidosis improved, although he did have persistent hypocalcemia, prompting several doses of calcium gluconate. Interestingly, despite fluid resuscitation, the patient’s blood urea nitrogen and Cr steadily worsened throughout his several days of admission. This was thought to be an intrarenal process, consistent with acute tubular necrosis.

For deep vein thrombosis prophylaxis, the patient was started on subcutaneous heparin. For the patient’s chronic medical conditions and cancer-related pain, we continued his home medications of levothyroxine and hydromorphone.

During his admission, the patient developed hemoptysis and melena, with a corresponding downtrend in his hemoglobin. He denied any previous history of melena but stated that his hemoptysis was due to esophageal scarring from his past medical history of tonsillar cancer and radiation. His heparin was discontinued, and the patient was placed on proton pump inhibitors for concern of gastrointestinal bleeding. However, with a thorough workup revealing no clear source of bleeding, and with the patient’s bleeding symptoms steadily improving, further intervention was not recommended.

Unfortunately, although there was improvement in the patient’s electrolytes, his cancer continued to progress, developing anemia and hypotension in the setting of hemoptysis and melena. After discussing with oncology, nephrology, and the patient’s family, it was determined that the patient was not a candidate for curative chemotherapy, and he was subsequently transitioned to palliative care with comfort measures only.

## Discussion

Spontaneous tumor lysis is a serious oncologic emergency. The authors propose that although spontaneous TLS is more common in hematological malignancies, it may also occur in the setting of solid tumors, particularly if there is metastasis present, causing high cell proliferation and turnover. As with our patient, the majority of similar case reports describe TLS in metastatic cancers; it appears to be uncommon for TLS to develop in the setting of solitary solid tumors [5-9].

TLS is defined by the Cairo and Bishop diagnostic criteria [1]. Diagnosis requires at least two laboratory findings in addition to at least one clinical finding (Table 2).

**Table 2:** Cairo and Bishop diagnostic criteria for TLS. *: 6.5 mg/dL in the pediatric population. TLS: tumor lysis syndrome

Laboratory TLS findings	Clinical TLS findings
Uric acid that increases at least 25% from baseline or is greater than or equal to 8.0 mg/dL	Creatinine greater than 1.5 times the upper limit of the age-adjusted reference range
Potassium that increases at least 25% from baseline or greater than or equal to 6.0 mEq/L	The presence of seizures
Phosphorus that increases at least 25% from baseline or greater than or equal to 4.5 mg/dL*	Cardiac arrhythmia or sudden death
Calcium that decreases at least 25% from baseline or less than or equal to 7.0 mg/dL	-

Based on these requirements, our patient met the diagnostic criteria for TLS. That being said, it is important to note that our patient’s lactate dehydrogenase (LDH) levels were only mildly elevated at 259 U/L, which is a deviation from most classic TLS cases. Indeed, previous reports of spontaneous TLS in the setting of pancreatic cancer were associated with much higher LDH ranges, such as 2,350 to 3,410 U/L [7]. However, other papers on TLS in recently diagnosed pancreatic carcinoma did not report LDH levels [12]. Unfortunately, our team was unable to obtain repeat LDH measurements for comparison before the patient was transferred to hospice.

Although our patient’s LDH level was peculiar in the context of his overall clinical picture, LDH is not part of the diagnostic criteria for TLS. In a patient who meets the criteria for TLS, the absence of certain features such as severe LDH elevation should not deter providers from pursuing adequate treatment. Because spontaneous TLS has a poor prognosis, with increased mortality and morbidity, we believe that a high index of suspicion is essential for early diagnosis and treatment initiation.

## Conclusions

It is imperative that future providers always consider the possibility of TLS when there is metabolic derangement in a cancer patient, for both hematological and solid tumors. Early detection and treatment of this condition may improve prognosis and/or comfort for the patient. This case demonstrates an example of successfully managed TLS in a patient with pancreatic adenocarcinoma. Our patient’s metabolic panel stabilized with continuous D5W, rasburicase, sevelamer, and calcium gluconate. Although the underlying disease could not be prevented from progressing, the treatment of his oncologic emergency likely afforded the patient much-needed additional time with loved ones as he transitioned to hospice care. Critical care and inpatient hospitalists should heed the importance of quickly identifying and managing TLS for the benefit of both the patient and their families.
